# Vacuum-assisted closure for chest wall reconstruction infection caused by *Streptococcus mitis* after surgery of lung cancer: a case report

**DOI:** 10.1186/s40792-024-01828-7

**Published:** 2024-01-31

**Authors:** Nozomu Motono, Takaki Mizoguchi, Masahito Ishikawa, Shun Iwai, Yoshihito Iijima, Hidetaka Uramoto

**Affiliations:** Department of Thoracic Surgery, Kanazawa Medical University, 1-1 Daigaku, Uchinada, Ishikawa 920-0293 Japan

**Keywords:** Vacuum-assisted closure, Chest wall reconstruction infection, Lung cancer, Polytetrafluoroethylene, *Streptococcus mitis*

## Abstract

**Background:**

Among a cohort of patients who underwent chest wall resection and reconstruction by rigid prosthesis, 6% required removal of the prosthesis, and in 80% of these cases the indication for prosthesis removal was infection. Although artificial prosthesis removal is the primary approach in such cases of infection, the usefulness of vacuum-assisted closure (VAC) has also been reported.

**Case presentation:**

A 64-year-old man with diabetes mellitus underwent right middle and lower lobectomy with chest wall (3rd to 5th rib) resection and lymph node dissection because of lung squamous cell carcinoma. The chest wall defect was reconstructed by an expanded polytetrafluoroethylene (PTFE) sheet. Three months after surgery, the patient developed an abscess in the chest wall around the PTFE sheet. We performed debridement and switched to VAC therapy 2 weeks after starting continuous drainage of the abscess in the chest wall. The space around the PTFE sheet gradually decreased, and formation of wound granulation progressed. We performed wound closure 6 weeks after starting VAC therapy, and the patient was discharged 67 days after hospitalization.

**Conclusions:**

We experienced a case of chest wall reconstruction infection after surgery for non-small cell lung cancer that was successfully treated by VAC therapy without removal of the prosthesis. Although removal of an infectious artificial prosthesis can be avoided by application of VAC therapy, perioperative management to prevent surgical site infection is considered essential.

## Background

The proportion of operable non-small cell lung cancer (NSCLC) invading the chest wall is about 5% [1]. After surgical removal of involved structures, reconstructive procedures using a rigid prosthesis, such as absorbable synthetic polyglactin mesh or expanded polytetrafluoroethylene (PTFE) sheet, are often necessary to maintain or re-establish thoracic cage stability and function [2]. Among a cohort of patients who underwent chest wall resection and reconstruction by rigid prosthesis, 6% required prosthesis removal [3]. Furthermore, in 80% of patients with indication for prosthesis removal, the etiology was related infection. Although prosthesis removal is indicated in cases of such prosthetic infection, the usefulness of vacuum-assisted closure (VAC) therapy for vascular graft infection has also been reported [4, 5]. VAC therapy was reported to contribute to removal of fluid material and wound healing by accelerated granulation growth, increased angiogenic factor production, and improved fibrin deposition [6].

We herein report a case of chest wall reconstruction infection after surgery for NSCLC that was successfully treated by VAC therapy without removal of the prosthesis.

## Case presentation

**Fig. 1:**
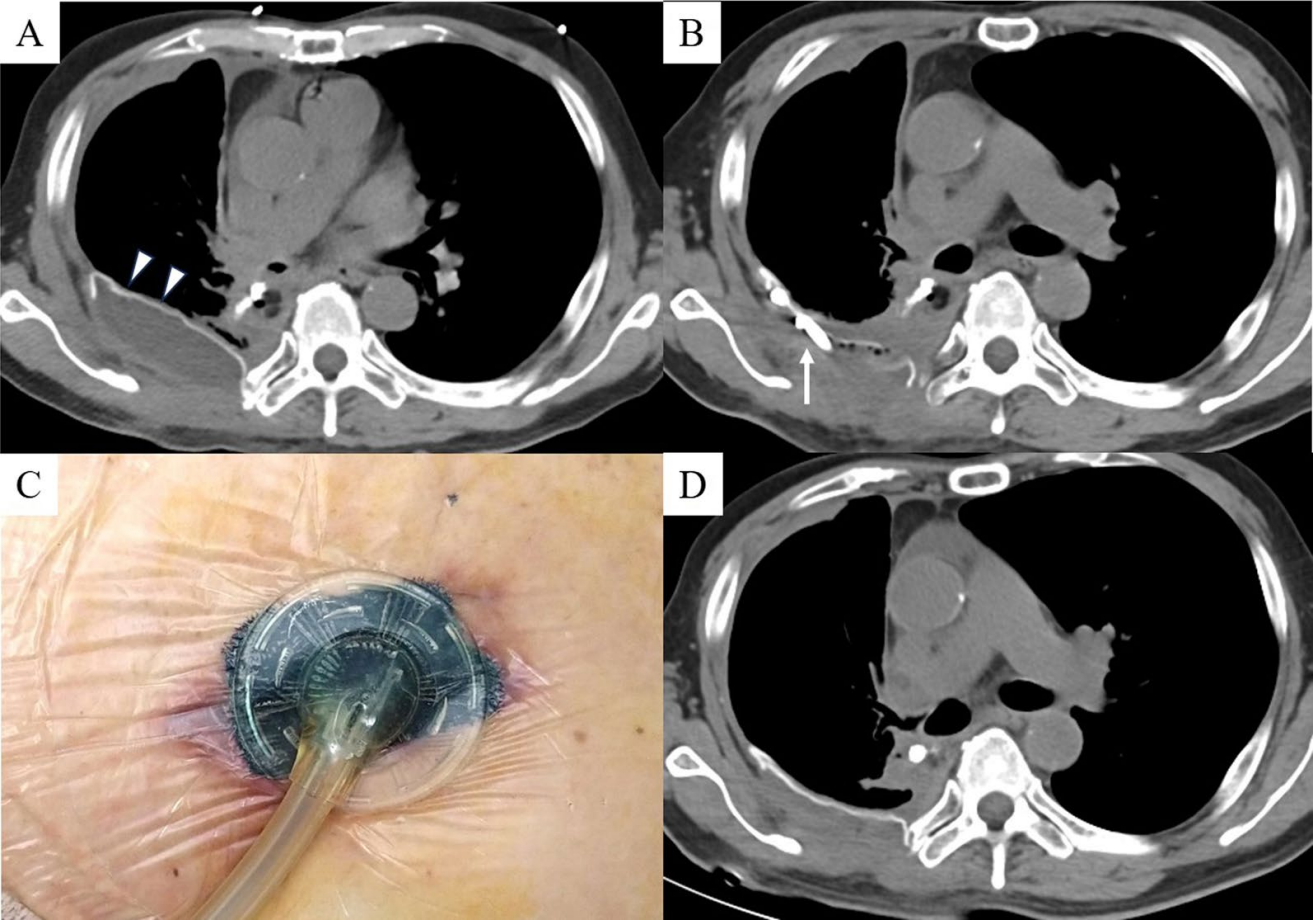
**A** Chest computed tomography (CT) showed fluid collection in the chest wall around chest wall reconstruction by expanded polytetrafluoroethylene (PTFE) sheet (white arrowheads). **B** The fluid collection in the chest wall was decreased by continuous drainage (white arrow). **C** Vacuum-assisted closure (VAC) therapy was performed for chest wall infection. **D** The space around the PTFE sheet had decreased on chest CT after VAC therapy

**Fig. 2:**
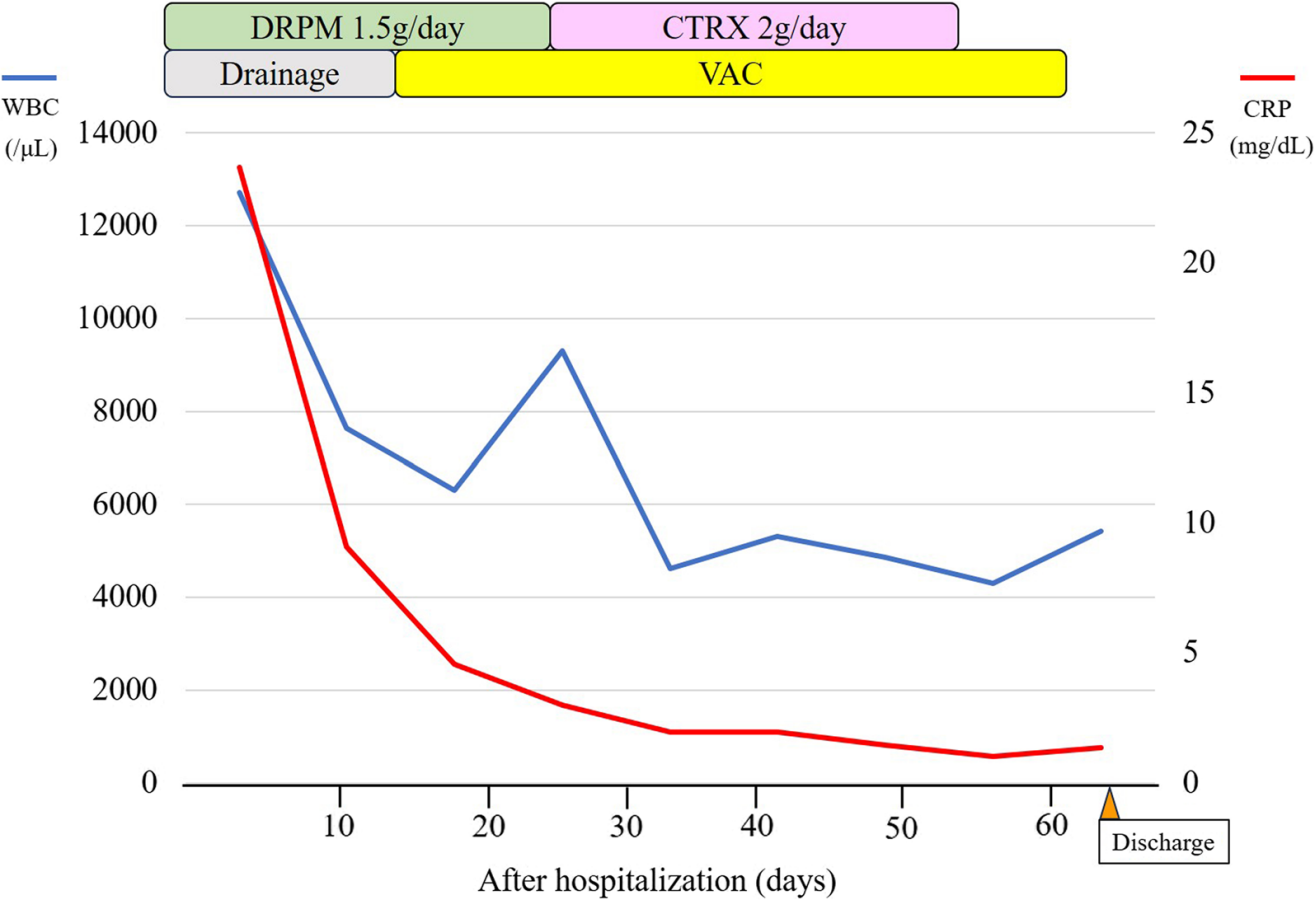
Treatment progress chart. *DRPM* doripenem, *CTRX* ceftriaxone

**Fig. 3:**
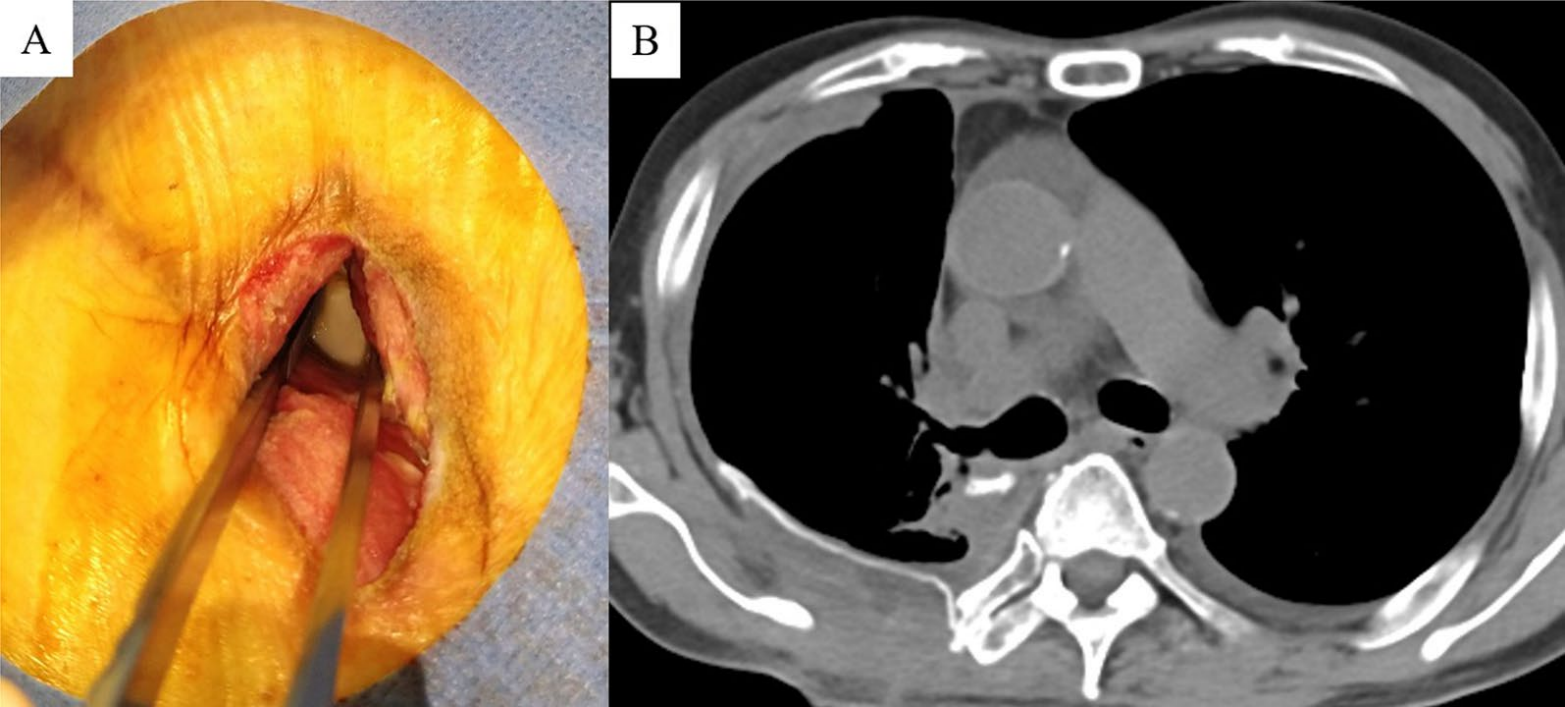
**A** Formation of wound granulation progressed after VAC therapy. **B** There was no renewed increase in the space around the PTFE sheet on chest CT

A 64-year-old man with diabetes mellitus who received chemoradiotherapy for small cell lung cancer of right lower lobe of lung 20 years previously underwent right middle and lower lobectomy with chest wall (3^rd^-to-5th rib) resection and lymph node dissection because of lung squamous cell carcinoma. The chest wall defect was reconstructed by an expanded PTFE sheet. Three months after surgery, the patient was referred our hospital with high fever and consciousness disorder. Laboratory findings showed white blood cell (WBC) count of 12,710/μL and C-reactive protein (CRP) level of 23.68 mg/dL, and chest computed tomography (CT) showed fluid collection in the chest wall around the PTFE sheet (Japan Gore-Tex Inc., Tokyo, Japan) reconstruction (Fig. 1A). Continuous drainage of the fluid collection in the chest wall (Fig. 1B) and administration of intravenous antibiotics (doripenem hydrate, 1.5 g/day) were initiated as empiric treatment, after which the WBC count and CRP level gradually decreased. Because bacterial culture of the wound revealed a small amount of *Streptococcus mitis* whereas blood cultures was negative of bacteria, we determined that these therapies would not completely cure the infection around the reconstruction. Furthermore, because we considered the risk of infection spreading into the thoracic cavity by removing the ePTFE sheet, we performed debridement and switched to VAC (3 M Japan Limited, Tokyo, Japan) therapy without removing the PTFE sheet (Fig. 1C) 2 weeks after starting drainage. We changed the VAC system every 2–3 days and de-escaleted to ceftriaxone sodium hydrate (2 g/day) which was revealed by drug susceptibility test based on clinical and laboratory standards institute as minimal inhibitory concentration less than 0.12, whereby the CRP level gradually decreased and the space around the PTFE sheet was observed to decrease on chest CT (Fig. 1D). Although administration of intravenous antibiotics was terminated 4 weeks after starting VAC therapy, the CRP level did not rise (Fig. 2), formation of wound granulation progressed, and the area around the PTFE sheet remained clean (Fig. 3A). We performed wound closure 6 weeks after starting VAC therapy, the CRP level remained stable, and the patient was discharged home 67 days after hospitalization. The space around the PTFE sheet did not show any renewed increase on chest CT at 4 weeks after wound closure (Fig. 3B), and a month has passed without no recurrence since the chest CT.

## Discussion

Since the introduction of VAC therapy in 1997 [7], its application for the treatment of intrathoracic infections and chest wounds has been reported [8–11]. The expected aim of VAC therapy is to control the infection site, increase the granulation tissue and tissue blood supply, and reduce the total volume of the cavity by intermittent negative pressure [12]. Local infection after chest wall resection and reconstruction has been reported as between 3.3% and 23%, with between 18 and 100% of these cases yielding removal of the prosthetic material [13, 14]. Because we considered the risk of infection spreading into the thoracic cavity by removing the PTFE sheet, we performed debridement and switched to VAC therapy without removing the PTFE sheet. Although there are no randomized trial of VAC therapy and removal of the prosthetic material for infection after chest wall resection and reconstruction, there has been reported the randomized trial of VAC therapy in the treatment of diabetic foot wounds [15]. In this report, compared to standard moist wound therapy, VAC therapy resulted in lower resource utilization and a greater proportion of patients obtaining wound healing at a lower overall cost of care whereas there was no difference in hospital stay. Randomized controlled trials should be required to determine whether VAC therapy or removal of the prosthetic material is more effective for infection after chest wall resection and reconstruction in terms of cost, length of treatment, and effectiveness.

*Streptococcus mitis* (*S. mitis*) has been individually identified as a part of the normal oropharyngeal flora, and has been mostly reported in cases of bacterial endocarditis and lung abscess through the bloodstream or respiratory tract [16–19]. On the other hand, there are a few rare cases of urinary tract infection, spondylodiscitis, and empyema caused by *S. mitis* [20–22]. Although the cause of infection around PTFE sheet in the present case was unclear because of no odontogenic infection and negative of the blood culture was negative, it is considered an extremely rare case.

New materials such as titanium plates, cryopreserved graft, and acellular collagen matrices have been reported to reduce the need for removal of the prosthesis from an infected area in comparison with conventional materials such as methyl methacrylate, PTFE, polypropylene, and polyglactin [14]. However, there are reports of treatment and cure by VAC therapy without removal of infected PTFE or Dacron vascular graft [4, 5]. As in the present case, removal of the artificial prosthesis at the infection site of chest wall reconstruction might be avoided because of the beneficial effect of VAC therapy.

Mean interval time between the first and second operations was reported as 4 months in one report [14] and as long as 15.4 months in another [3]. The interval time between the first and second operations in the present case was 3 months, so the infection is considered to be relatively early. Although the cause of postoperative local infection around the PTFE sheet was unclear, it was reasoned that there was no infection within the thoracic cavity and that the infection only affected the area outside the PTFE sheet. The risk factors of surgical site infection (SSI) are reported to be diabetes mellitus, foreign material, duration of operation, and postoperative hyperglycemia, some of which apply to the current case [23]. In our patient there was severe adhesion overall within the thoracic cavity, considered to be due to chemoradiotherapy for small cell lung cancer 20 years previously, and the operation time of 6.5 h was considered to be related to the occurrence of SSI. Based on a review article, it was thought necessary to make efforts to maintain intensive blood glucose values between 110 and 150 mg/dL as a postoperative prevention strategy [23].

## Conclusions

We experienced a case of chest wall reconstruction infection after surgery for NSCLC that was successfully treated by VAC therapy without removal of the prosthesis. Although removal of an infectious artificial prosthesis might be avoided by the use of VAC therapy, perioperative management to prevent SSI is considered extremely important.

## Data Availability

Not applicable.

## Abbreviations

VAC: Vacuum-assisted closure
NSCLC: Non-small cell lung cancer
PTFE: Polytetrafluoroethylene
CRP: C-reactive protein
WBC: White blood cell
CT: Computed tomography
SSI: Surgical site infection
